# Teicoplanin Suppresses Vegetative *Clostridioides difficile* and Spore Outgrowth

**DOI:** 10.3390/antibiotics10080984

**Published:** 2021-08-15

**Authors:** Suvash Chandra Ojha, Matthew Phanchana, Phurt Harnvoravongchai, Surang Chankhamhaengdecha, Sombat Singhakaew, Puey Ounjai, Tavan Janvilisri

**Affiliations:** 1 Graduate Program in Molecular Medicine, Faculty of Science, Mahidol University, Bangkok 10400, Thailand; suvash_ojha@swmu.edu.cn; 2 Department of Infectious Diseases, The Affiliated Hospital of Southwest Medical University, Luzhou 646000, China; 3 Department of Molecular Tropical Medicine and Genetics, Faculty of Tropical Medicine, Mahidol University, Bangkok 10400, Thailand; matthew.pha@mahidol.edu; 4 Department of Biology, Faculty of Science, Mahidol University, Bangkok 10400, Thailand; phurt.har@mahidol.edu (P.H.); surang.cha@mahidol.ac.th (S.C.); sombat.sin@mahidol.ac.th (S.S.); puey.oun@mahidol.edu (P.O.); 5 Department of Biochemistry, Faculty of Science, Mahidol University, Bangkok 10400, Thailand

**Keywords:** teicoplanin, *Clostridioides difficile*, spore, antibiotics

## Abstract

In recent decades, the incidence of *Clostridioides difficile* infection (CDI) has remained high in both community and health-care settings. With the increasing rate of treatment failures and its ability to form spores, an alternative treatment for CDI has become a global priority. We used the microdilution assay to determine minimal inhibitory concentrations (MICs) of vancomycin and teicoplanin against 30 distinct *C. difficile* strains isolated from various host origins. We also examined the effect of drugs on spore germination and outgrowth by following the development of OD_600_. Finally, we confirmed the spore germination and cell stages by microscopy. We showed that teicoplanin exhibited lower MICs compared to vancomycin in all tested isolates. MICs of teicoplanin ranged from 0.03–0.25 µg/mL, while vancomycin ranged from 0.5–4 µg/mL. Exposure of *C. difficile* spores to broth supplemented with various concentrations of antimicrobial agents did not affect the initiation of germination, but the outgrowth to vegetative cells was inhibited by all test compounds. This finding was concordant with aberrant vegetative cells after antibiotic treatment observed by light microscopy. This work highlights the efficiency of teicoplanin for treatment of *C. difficile* through prevention of vegetative cell outgrowth.

## 1. Introduction

*Clostridioides difficile*, previously known as *Clostridium difficile*, is a gram-positive anaerobic spore-forming bacterium. It accounts for about 20–25% of antibiotic associated diarrhea [1,2] and almost all cases of pseudomembranous colitis [3]. *C. difficile* infection (CDI) normally occurs after antibiotic administration, especially ampicillin and amoxicillin, cephalosporins, clindamycin, fluoroquinolones, and meropenem [3,4]. Studies have suggested that gut microbiota dysbiosis after antibiotic treatment allows colonization and growth of *C. difficile* [5]. CDI can cause clinical manifestations ranging from asymptomatic to severe diarrhea, pseudomembranous colitis, bowel perforation, and multi-organ dysfunction [6]. Ultimately, CDI can be fatal, mostly in older patients [7]. The total CDI incidence has decreased in the US according to the Center for Disease Control and Prevention (CDC) [8]. Although the number of cases is not on the rise, *C. difficile* is classified as a pathogen posing an urgent threat due to antibiotic resistance [9].

Treatment for CDI is now limited to a few antibiotics including fidaxomicin and vancomycin according to the new guideline by the Infectious Diseases Society of America (IDSA) and the Society for Healthcare Epidemiology of America (SHEA) [10]. Metronidazole, which was suggested as a first line, is now recommended only when fidaxomicin or vancomycin is not available or is limited, owing to its inferiority to vancomycin and fidaxomicin, higher recurrence rate, and neurotoxicity in prolonged or repeated use [10,11]. Additionally, treatment failures have been reported for most regimens, mostly due to the recurrence of *C. difficile*, hence new antibiotics for CDI are of utmost importance [12].

Developing new drugs is a costly and time-consuming process. Therefore, drug repurposing or repositioning has come under the limelight in pharmaceutical research in recent years as it can cut down the development process to minimal [13,14]. Teicoplanin, a mixture of glycopeptide antibiotics, belongs to the same class as vancomycin, distinguished by glucosamine as the basic sugar and the presence of aliphatic acid residues. It binds to the terminal D-Ala-D-Ala sequence of peptides forming the bacterial cell wall and, by sterically hindering the transglycosylation reaction, inhibits the formation of peptidoglycan by an intracellular accumulation of UDP-*n*-acetylmuramyl-pentapeptide [15]. Teicoplanin exhibits great activity against multiple gram-positive bacteria, which fail for other regimens [16], including *C. difficile* [17]. Even though the activity of teicoplanin against vegetative cell *C. difficile* is well documented [17,18,19], no experimental evidence has been presented so far for spore and outgrowth of *C. difficile*. We investigated the effect of teicoplanin on spore germination and outgrowth in *C. difficile* isolates from different host origins and compared it to its vancomycin counterpart. In addition, we also examined the cell stage alterations marked by staining the affinity of germinating cells following antibiotic treatment. The data presented here provide experimental evidence for the inhibitory effect of teicoplanin on germinating *C. difficile* cells.

## 2. Results

### 2.1. MICs of Teicoplanin on C. difficile

**Figure 1:**
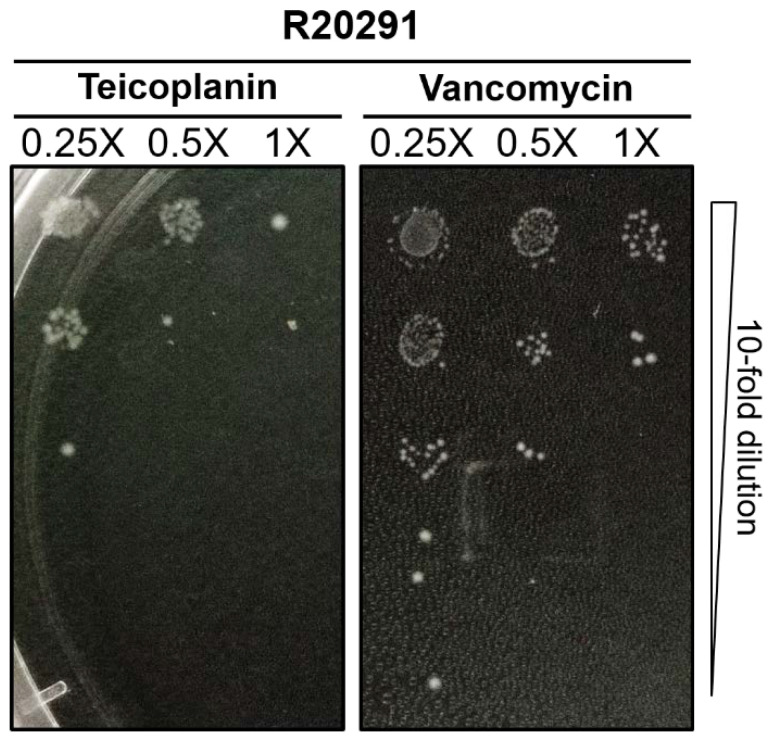
Antibacterial activity at sub-MICs of antibiotics using spot assay. A representative strain of *C. difficile* R20291 was attained to 0.1 OD_600_ in BHIS (brain heart infusion supplemented with 0.1% sodium taurocholate) medium supplemented with 0.25×, 0.5×, 1× MICs of teicoplanin or vancomycin. After 24-h incubation, cultures were 10-fold serially diluted and spotted onto BHIS agar plates, and plates were photographed following 24 h anaerobic incubation at 37 °C.

Minimum inhibitory concentrations (MICs) of teicoplanin and vancomycin against 30 *C. difficile* isolates obtained from various sources were determined by the broth microdilution method as described earlier [20]. All strains tested were sensitive to both antibiotics with MIC ranges for teicoplanin and vancomycin of 0.03–0.25 µg/mL and 0.5–4.0 µg/mL, respectively. Teicoplanin showed lower MICs among all strains tested (Table 1). We also compared the effect of teicoplanin at sub-MICs to vancomycin on selected strains. At sub-MICs, both antibiotics were at least 1-log less potent than at their respective MICs. However, teicoplanin at sub-MIC concentrations reduced the number of colonies more than that of vancomycin (Figure 1). The MICs of antimicrobial agents for each respective vegetative strain were used as a platform to evaluate the effect of these drugs on *C. difficile* spore germination and outgrowth. We also evaluated minimum bactericidal concentrations (MBC) of both teicoplanin, and vancomycin and the results revealed that the MBC of both antibiotics were at the concentration of 2× MIC.

### 2.2. Teicoplanin Does Not Inhibit C. difficile Spore Germination

**Figure 2:**
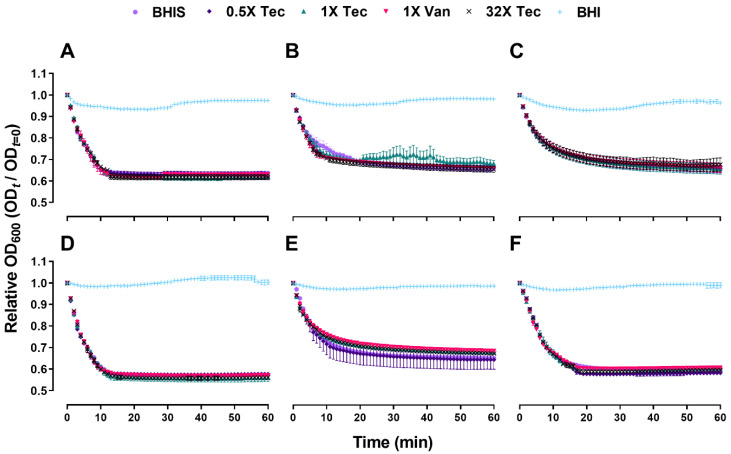
Teicoplanin and vancomycin do not inhibit spore germination. *C. difficile* spores were exposed to different concentration of antibiotics in relation to their respective MICs, and germination was followed by measuring loss of OD_600_ for 1 h at 1 min time interval. The growth control contained no antibiotic, and as a comparator, vancomycin was included. Data points represent the mean of the relative OD_600_ at the indicated time points normalized to *t* = 0 (control). All the experiments were performed in triplicates and error bars represent the standard errors. The results include the strains: (**A**) F101; (**B**) F102; (**C**) A125; (**D**) A126; (**E**) R20291; (**F**) H203. Circle (●), diamond (◆), triangle (▲), inverted tringle (▼), cross (×), and plus (+) denote the exposure to BHIS, 0.5× MIC teicoplanin, 1× MIC teicoplanin, 1× MIC vancomycin, 32× MIC teicoplanin, and BHI, respectively. Abbreviations: Tec = teicoplanin, Van = vancomycin, BHI = Brain heart infusion broth without 0.1% sodium taurocholate, BHIS = Brain heart infusion broth supplemented with 0.1% sodium taurocholate.

To determine the role of teicoplanin and vancomycin in its ability to block *C. difficile* spore germination, purified spores of 6 *C. difficile* strains were incubated for 1 h in the presence of BHIS supplemented with 0.5×, 1×, or 32× MIC of teicoplanin, and germination kinetics were monitored by observing the changes in OD_600_ of *C. difficile* cultures over a 1 h period. A drug-free control and 1× MIC of vancomycin were included as a comparator. As expected, *C. difficile* isolates germinated poorly in BHI broth without supplementation of 0.1% taurocholate. Nevertheless, teicoplanin and vancomycin did not inhibit spore germination at all concentrations tested (*p* > 0.05) (Figure 2), resulting in a significant drop of OD_600_. The initiation of spore germination under treatment conditions was comparable to the drug-free control over time based on ANOVA at each time point.

### 2.3. Teicoplanin Inhibits C. difficile Spore Outgrowth

**Figure 3:**
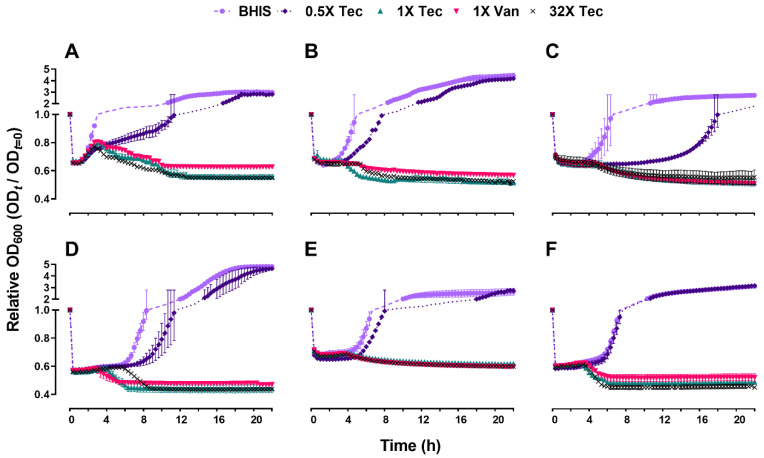
Teicoplanin and vancomycin at MIC inhibit spore outgrowth, while sub-MIC teicoplanin substantially delays outgrowth. Data points represent the mean of the relative OD_600_ at the indicated time points normalized to *t* = 0 (control). All the experiments were performed in triplicates and error bar represents the standard errors. The results include the strains: (**A**) F101; (**B**) F102; (**C**) A125; (**D**) A126; (**E**) R20291; (**F**) H203. Circle (●), diamond (◆), triangle (▲), inverted tringle (▼), and cross (×) denote the exposure to BHIS, 0.5× MIC teicoplanin, 1× MIC teicoplanin, 1× MIC vancomycin, and 32× MIC teicoplanin, respectively. Abbreviations: Tec = teicoplanin, Van = vancomycin, BHIS = Brain heart infusion broth supplemented with 0.1% sodium taurocholate. The dashed and dotted lines represent the data trend between the Y-axis break.

Since both teicoplanin and vancomycin did not have either a positive or negative influence on the initiation of spore germination, we next evaluated the outgrowth to vegetative cells by monitoring the change in OD_600_, to examine whether the later stage of germination would be affected. As expected, under the influence of 0.5× MIC of teicoplanin, growth differences were observed in contrast to the untreated control, although not significantly different (Figure 3). Sub-inhibitory concentrations of teicoplanin appeared to delay the onset of spore outgrowth and increased the time until the stationary phase was reached. However, they did not affect the outcome or the later stage of spore outgrowth. The outgrowth of spores in all tested strains exposed to a minimum of MIC of drugs was significantly inhibited compared to the drug-free control (*p* < 0.0001).

**Figure 4:**
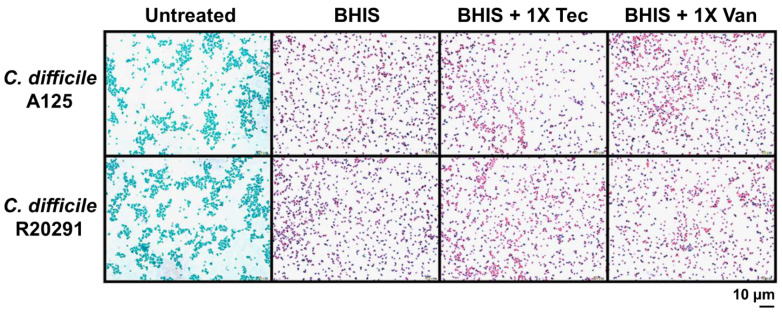
Teicoplanin and vancomycin at their respective MICs do not inhibit initiation of spore germination as revealed by differential staining. *C. difficile* spores were incubated with 1× MIC of either teicoplanin or vancomycin supplemented with BHIS medium for 3 h. Germinated cells were stained by Wirtz–Conklin staining (5% malachite green/0.5% safranin). Spores were greenish-blue spheres and germinated cells appeared to be pink/purple spheres. All the micrographs were taken at a magnification of 1000×. All the experiments were repeated in triplicates to ensure the reproducibility of the results. Abbreviations: Tec = teicoplanin, Van = vancomycin, BHIS = Brain heart infusion broth supplemented with 0.1% sodium taurocholate.

**Figure 5:**
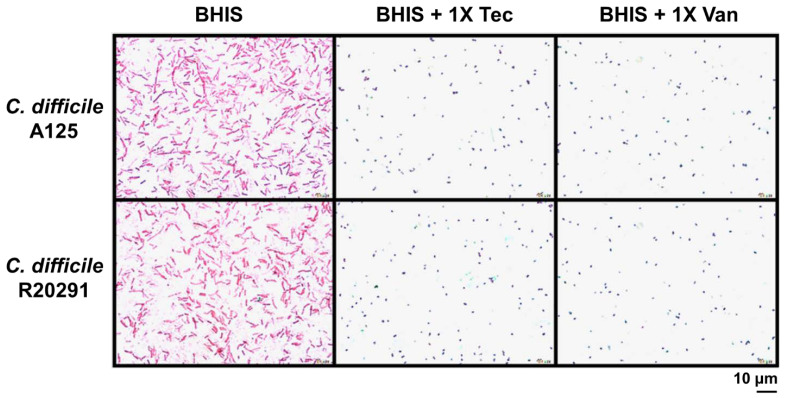
Antibiotic treatments limit the outgrowth of germinated *C. difficile* spores and prevent outgrowth to vegetative cells. *C. difficile* spores were incubated with BHIS medium supplemented with either 1× MIC of teicoplanin or vancomycin for 24 h. Germination inhibition and outgrowth were marked by Wirtz–Conklin staining. Vegetative cells were long filamentous and stained pink/purple, germinated cells were pink/purple spheres, outgrowths were pink/purple/faintly stained, blunted ends or extrusion from germinated cell. All the micrographs were taken at a magnification of 1000×, 5 fields counted, and repeated in triplicates to ensure the reproducibility of the results. Abbreviations: Tec = teicoplanin, Van = vancomycin, BHIS = Brain heart infusion broth supplemented with 0.1% sodium taurocholate (as negative control).

**Figure 6:**
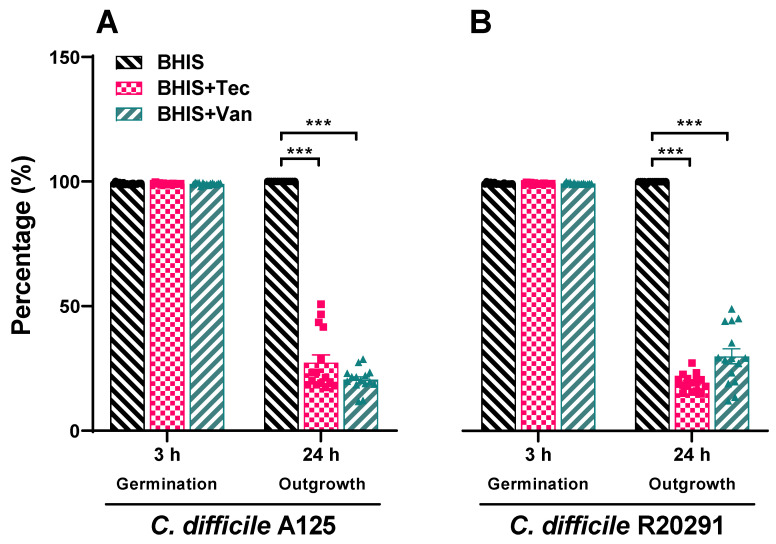
Teicoplanin and vancomycin significantly reduce outgrowth in *C. difficile* (**A**) A125 and (**B**) R20291 as determined by differential staining assay. *C. difficile* spores were incubated with BHIS medium alone or medium supplemented with 1× MIC of either teicoplanin or vancomycin for 24 h, then thoroughly washed and stained with a Wirtz–Conklin stain. All the micrographs were taken at a magnification of 1000×. Relative percentage of germinated spore and vegetative cells were calculated based on the count of five different fields by CellSens Dimension software. Bar graphs represent geometric means of three independent experiments and error bars represent the standard errors. Abbreviations: Tec = teicoplanin, Van = vancomycin and *** denotes *p*-value < 0.0001.

To further investigate the alteration under the influence of antibiotics on spore germination and outgrowth, we included strains that were the least and the most susceptible to teicoplanin, R20291 and A125, respectively. We next performed Wirtz–Conklin staining of untreated spores or spores treated with 1× MIC of teicoplanin or vancomycin following 3 h incubation in the growth medium. The number of spores estimated by visual inspection using light microscopic analysis revealed that >98% of spores germinated in both antibiotic treatment groups, which was not significantly different from the untreated control (Figure 4 and Figure 6). The control spores appeared as greenish-blue spheres, and the germinated cells appeared as pink spheres without shape alteration and no bacilli were detected. However, the inhibition of outgrowth by these antibiotics was more evident as determined by microscopic analysis (Figure 5), confirming that antibiotic exposure did inhibit outgrowth to vegetative cells. After 24 h incubation in a growth medium supplemented with 1× MIC of antibiotics, spores treated with both antibiotics appeared to lose their ability to change to bacillus vegetative cells. Spores were stained pink/purple or faintly stained with spherical or blunted rounded ends and showed structural degeneration and clear extrusion from the spore germinated structure (Figure 5). This suggested that the outgrowth was inhibited at the initial stage of germination. Both antibiotics at 1× MIC inhibited up to 80% of spore outgrowth to vegetative cells (Figure 5 and Figure 6). Statistical analysis suggested that teicoplanin and vancomycin at their respective MICs inhibited spore outgrowth significantly (*p* < 0.001) when compared to untreated control. However, the effect of teicoplanin and vancomycin on spore outgrowth was not significantly different (Figure 6).

## 3. Discussion

CDI continues to be a major nosocomial pathogen and a particular source of morbidity and mortality among elderly and immune suppressed individuals [21]. Treatment failures have been more evident recently, which has raised a serious concern for clinicians across multiple specialties. Hence, there is a medical need to explore potential therapeutic drugs with improved properties. Previous studies have encouraged the use of teicoplanin over commonly used antibiotics in CDI treatment due to its longer half-life, lower relapse rate, relatively uncommon nephrotoxicity or cytotoxicity, and lack of requirement for routine serum monitoring [18,19,22,23] A study by Wenisch et al. (1996) also claimed to have 100% cure rate with the use of teicoplanin in patients endoscopically confirmed with pseudomembrane colitis [19]; however, direct comparisons with vancomycin are difficult because of inherent differences between studies.

As teicoplanin is fast acting at low concentrations and has poor absorption in the gut [24], it is incontestably an effective antimicrobial agent for the control of pathogens in the gut, including *C. difficile,* without permitting spore formation [25,26]. The activity of teicoplanin was at least 8–16 fold more potent than that of vancomycin, which was consistent with the studies done by Kunishima [27]. A set of 6 distinct target isolates obtained from humans, animals and food were used to account for the variation in the *C. difficile* spore germination and outgrowth under the influence of these antimicrobial agents. Examining the 0.5×, 1× or 32× of teicoplanin or 1× MIC of vancomycin on *C. difficile* spore germination revealed that none of the antimicrobial agents affected the initiation of germination compared to the drug-free control. At MIC or above, we observed that the outgrowth was inhibited by these antimicrobial agents. This is predictable as spores lose their dormancy upon germination, resume metabolism at the core region and subsequently an outgrowth begins by synthesizing new cell wall peptidoglycan [28,29]. The germinated spores are vulnerable to these glycopeptide antibiotics, which inhibit spore outgrowth. Although teicoplanin is functionally similar to vancomycin, its potent activity at relatively low concentration on vegetative cells and spore outgrowth may contribute to the lower recurrence rate in clinical trials [19,30,31,32,33]. Furthermore, transition from spores to vegetative cells is important and involves various metabolic changes. There are reports showing that some antibiotics can inhibit vegetative cells but not outgrowth [34]. Certain antibiotics inhibit both spore outgrowth and vegetative cells but at different concentrations, implying that there are underlying differences between these stages [35]. 

At sub-inhibitory concentration of teicoplanin, late growth had begun for most strains, which took an extended duration to reach their stationary phase; this could be due to the stress generated by the antibiotic at an early stage of spore germination. 

To investigate further spore germination and outgrowth by light microscopy, we included 2 strains that were the least and the most susceptible to teicoplanin. Wirtz–Conklin staining displayed a clear distinction between different stages of spore germination following antibiotic treatment or untreated spores, which agreed with the studies done by Hamouda [36]. Untreated spores were dormant in structure and acquired greenish blue color following Wirtz–Conklin staining instead of pink/purple color spheres or rods [21,36,37]. Incubating spores with growth medium for 3 h displayed initiation of germination without complete transition to bacilli, where they appeared as pink spheres. This change in their staining affinity is associated with the initiation of germination in the presence of growth medium without complete outgrowth to the filamentous vegetative bacilli. The variability in spore germination appeared due to the asynchrony in spore population germination following exposure to spore germinants, which followed studies done by Moir [38]. Following complete germination, spores were transformed into filamentous vegetative cells that were stained purple rods. However, spores following treatment with antibiotics did not develop into bacilli for up to 24 h. The inhibitory action of those antibiotics affected their outgrowth to vegetative cells, which appeared as spheres or short rods with blunted ends, supporting the notion of being sporostatic agents.

## 4. Materials & Methods

### 4.1. C. difficile Strains and Growth Conditions

A total of 30 *C. difficile* isolates obtained from various sources, including food, animal, and human were used in this study (Table 1) [39]. *C. difficile* strain R20291 was kindly provided by Prof Nigel Minton, University of Nottingham. As described previously [40,41], all *C. difficile* strains were grown at 37 °C in an anaerobic workstation (85% N_2_, 10% H_2_, and 5% CO_2_; Don Whitley Scientific, UK) in the brain heart infusion supplemented with 0.1% (*w*/*v*) sodium taurocholate, 0.1% L-cysteine, and 5 mg/mL yeast extract broth or agar (BHIS).

### 4.2. Spore Preparation

A total of 6 strains of *C. difficile* obtained from various sources were included for spore purification. Briefly, a single colony was inoculated into BHIS broth and incubated overnight at 37 °C. A 100-µL aliquot of overnight culture was spread onto BHIS agar, supplemented with 250 µg/mL cycloserine and 8 µg/mL cefoxitin, and incubated anaerobically at 37 °C for 10 days to allow efficient sporulation. Sporulation-induced lawns were harvested in 1 mL sterile distilled water (dH_2_O) using cell scrapers. The suspension was then centrifuged at 5000× *g* for 15 min and washed 5 times with sterile dH_2_O. To inactivate viable vegetative cells, spore suspensions were then treated with 0.3 mg/mL proteinase K at 37 °C for 2 h with gentle shaking, followed by incubation at 65 °C for 1 h. Subsequently, spore suspensions were washed 5 additional times to remove any residuals from proteinase K. Purified spores were examined by phase-contrast microscopy to ensure that they were free of vegetative cells and debris, and subsequently stored at 4 °C.

### 4.3. Antimicrobial Assay

To determine minimal inhibitory concentrations (MICs), a single colony from overnight culture was resuspended in 5 mL BHIS broth and incubated anaerobically for 12 h. Next day, 100 µL of aliquots were transferred to a new BHIS broth and incubated for another 6 h to minimize spore carryover and dilute the pre-formed toxin effect. The diluted vegetative cell suspension (100 µL) was aliquoted to the wells of flat-bottomed 96-well plates containing equal volumes of BHIS medium supplemented with defined concentrations of antibiotics, with the initial inoculum concentration maintained at OD_600_ of 0.6. An antibiotic-free control was included in each experiment. After 24 h incubation, the plates were measured for OD_600_ as an indicator of bacterial growth using a microplate reader (Tecan, Switzerland). MIC is defined by the concentration that has no visible growth. These assays were repeated at least 3 times to ensure reproducibility of the results. To compare the effect of sub-inhibitory concentrations of teicoplanin and vancomycin, *C. difficile* was exposed to 0.25×, 0.5×, and 1× MIC values, and was serially diluted before stamping on to BHIS plate, then incubated anaerobically for 24 h.

Minimal bactericidal concentration (MBC) was performed as previously described [42]. Briefly, the assay plate containing various concentrations of antibiotics was inoculated with bacterial suspension as per MIC and incubated for 24 h, then the bacterial suspensions around MIC value were transferred to the BHIS plate by the stamping technique and incubated for 24 h. MBC is defined by the concentration where no bacterial colony was observed on the BHIS plate. 

### 4.4. Spore Germination and Outgrowth

Spore suspensions were heat activated at 65 °C for 30 min, vortexed to obtain a homogenous suspension and checked for clumping by microscopy. The time-kill kinetics of teicoplanin against 6 *C. difficile* strains were performed at the 0.5×, 1×, 2×, and 32× MIC of antibiotics supplemented in BHIS medium, with the final inoculum concentration maintained at OD_600_ of 0.6. Spore germination was followed anaerobically at 37 °C by measuring the loss of OD_600_ at 1 min time intervals for 1 h using a microplate reader. Reduction of OD_600_ reflects spore germination as it changes with the refractility of the spore coat [43]. Following germination, the differences in spore outgrowth efficiency were recorded by measuring OD_600_ at 20 min time intervals for 22 h using the same protocol. The ratio of the OD_600_ at time t and the control (t = 0) was then plotted against time. A drug-free control, and as a comparator, the spore suspensions treated with 1× MIC of vancomycin were included for every strain tested.

### 4.5. Wirtz–Conklin Staining 

The staining protocol for differentiation of spores from germinating and vegetative cells was adapted from Hamouda [36]. The spore suspension was anaerobically incubated at 37 °C for 3 h or for 24 h with BHIS medium alone or medium supplemented with MIC concentrations of antibiotics. Following incubation, the spore suspension was washed thoroughly 5 times with sterile distilled water and resuspended with 15 µL dH_2_O (OD_600_~0.1). Five microliters of spore suspension were transferred to a clean glass slide and smeared uniformly. Henceforth, slides were air dried, and heat fixed and stained with Wirtz–Conklin stain. The specimen was visualized under oil immersion objective of a light microscope (Olympus BX53, Tokyo, Japan). Five fields of each slide were imaged from 3 independent biological replicates, counted by using software CellSens Dimension software version 1.11 (Olympus Software, Imaging System, Hamburg, Germany). Based on their color differences, relative percentages of germinated spores and vegetative cells were calculated. The percentage of spores in each image was also calculated as [Number of spores/Total number of cells (spores + vegetative cells)] × 100.

### 4.6. Statistical Analysis

All data presented were of at least 3 independent experiments. Statistical analyses were performed by the nonparametric one-way analysis of variance (ANOVA), using GraphPad Prism software (GraphPad Software Inc., La Jolla, CA, USA) to compare each condition with the corresponding controls. *p*-values less than 0.05 indicated statistically significant difference.

## 5. Conclusions

In conclusion, our results indicated that teicoplanin could be a potential therapeutic drug for *C. difficile* due to its potent activity at low concentrations, as well as having pre-determined broad-spectrum activity against gram-positive anaerobes. Teicoplanin did not interrupt spore germination, but instead inhibited the outgrowth to vegetative cells from the germinated spore. Our data bridge the experimental gap on the effect of teicoplanin on spores as its effects on spore germination and outgrowth in *C. difficile* have not yet been reported. As most *C. difficile*-associated diseases are multifactorial, further in-depth studies, including many hypervirulent strains, animal models and human trials must be warranted to elucidate the therapeutic role of teicoplanin.

## Figures and Tables

**Table 1:** Minimal inhibitory concentrations for teicoplanin and vancomycin for 30 *C. difficile* strains.

Strains	Origin	MIC (µg/mL)	
		Teicoplanin	Vancomycin
F101	Food	0.06	2
F102	Food	0.06	2
F103	Food	0.125	2
F104	Food	0.125	2
A121	Animal	0.25	4
A122	Animal	0.125	2
A123	Animal	0.25	4
A124	Animal	0.25	4
A125	Animal	0.25	4
A126	Animal	0.125	2
R20291	Human	0.03	0.5
H201	Human	0.06	1
H203	Human	0.125	2
H204	Human	0.06	1
H205	Human	0.25	4
H206	Human	0.25	4
H207	Human	0.25	4
H208	Human	0.25	4
H209	Human	0.125	2
H210	Human	0.125	2
H211	Human	0.25	4
H212	Human	0.125	2
H213	Human	0.25	2
H214	Human	0.25	4
H215	Human	0.25	4
H216	Human	0.125	2
H217	Human	0.125	2
H218	Human	0.25	4
32g57	Human	0.125	2

## Data Availability

Data are contained within the article.
